# Heart Rate Variability Dynamics as Predictors of Functional Recovery and Mortality After Acute Ischemic Stroke

**DOI:** 10.3390/biomedicines13092217

**Published:** 2025-09-10

**Authors:** Oana Elena Sandu, Carina Bogdan, Adrian Apostol, Mihaela Adriana Simu, Victor-Dan Moga, Radu-Mihai Pecingina, Alexandru Covaciu, Viviana Mihaela Ivan

**Affiliations:** 1 Department VII, Internal Medicine II, Discipline of Cardiology, “Victor Babeş” University of Medicine and Pharmacy, Eftimie Murgu Sq. No. 2, 300041 Timişoara, Romania; oana.ciolpan@umft.ro (O.E.S.); adrian.apostol@umft.ro (A.A.); moga.victor@umft.ro (V.-D.M.); radu.pecingina@umft.ro (R.-M.P.); ivan.viviana@umft.ro (V.M.I.); 2 Doctoral School, “Victor Babeş” University of Medicine and Pharmacy, Eftimie Murgu Sq. No. 2, 300041 Timişoara, Romania; 3 Department of Neurology, “Victor Babeş” University of Medicine and Pharmacy Timișoara, Eftimie Murgu Sq. No. 2, 300041 Timișoara, Romania; simu.mihaela@umft.ro; 4 Department of Neurology, Clinical Emergency County Hospital “Pius Brînzeu” Timișoara, Bvd. Iosif Bulbuca No. 10, 300736 Timișoara, Romania; 5 M3 Department–Clinical and Medical-Surgical Disciplines, “George Emil Palade” University of Medicine, Pharmacy, Sciences and Technology from Târgu Mureș, 540139 Târgu Mureș, Romania; alexandru.covaciu92@gmail.com; 6 Department of Medical and Surgical Specialties, Faculty of Medicine, Transilvania University of Brasov, 500019 Brasov, Romania

**Keywords:** acute ischemic stroke, heart rate variability (HRV), autonomic dysfunction, functional outcome, modified Rankin Scale

## Abstract

**Background**: Autonomic dysfunction is commonly encountered after acute ischemic stroke (AIS) and may influence both functional recovery and survival. Heart rate variability (HRV) provides a non-invasive measure of autonomic balance, but its temporal evolution and prognostic significance in AIS remain insufficiently evaluated. **Methods**: In this prospective observational study, 148 AIS patients (mean age of 65.93 ± 9.19 years) underwent HRV assessment at baseline, one month, and three months follow-up, between January 2022 and October 2024. Time and frequency domain parameters, including Standard Deviation of NN intervals (SDNN), Low-Frequency (LF) power, High-Frequency (HF) power, and LF/HF ratio, were analyzed. Functional outcome was assessed using the modified Rankin Scale (mRS), with a good outcome defined as mRS ≤ 2. Multivariable logistic regression identified independent predictors of poor outcome (mRS > 2) at each time point. Mortality was recorded at one and three months, and potential predictors were evaluated. **Results**: Over three months, SDNN increased by 34.84% (*p* < 0.001), HF power rose by 22.26% (*p* < 0.001), LF power decreased by 21.61% (*p* < 0.001), and LF/HF ratio declined by 35.41% (*p* < 0.001), indicating a shift toward parasympathetic predominance. Higher SDNN correlated strongly with better functional status and was an important predictor of favorable outcome at all time points (*p* < 0.001). Higher LF/HF ratio predicted poor outcome at baseline (*p* < 0.01) and three months (*p* < 0.001). At three months, mortality reached 12.2%, with significant predictors including coronary artery disease (CAD), heart failure (HF), chronic kidney disease (CKD), and altered HRV parameters. **Conclusions**: Post-stroke recovery is characterized by the progressive restoration of autonomic balance, with higher SDNN and lower LF/HF associated with improved functional recovery and survival. HRV analysis offers valuable prognostic insight and may aid in risk stratification after AIS.

## 1. Introduction

Globally, stroke represents the second most common cause of mortality and a leading cause of long-term disability [1]. With increasing life expectancy and the rapid growth of the aging population, the incidence of stroke is projected to rise substantially due to population aging, with cases in adults over 60 years expected to nearly triple by 2050 [2]. These demographic trends highlight the need to better understand the factors influencing recovery and prognosis after stroke.

Post-stroke recovery is shaped by multiple factors, such as the initial stroke severity, the location and extent of brain injury, and the patient’s pre-existing health status. Among these, autonomic dysfunction has emerged as an important and often overlooked contributor that can compromise rehabilitation and negatively influence long-term functional outcomes [3,4]. The autonomic nervous system (ANS) regulates essential involuntary functions such as heart rate, blood pressure, respiration, and digestion. Stroke-related injury to central or peripheral autonomic pathways may disrupt the balance between sympathetic and parasympathetic activity, leading to sympathetic overactivity or parasympathetic withdrawal [5].

HRV provides a noninvasive measure of ANS function, reflecting fluctuations in the time intervals between consecutive heartbeats [6]. HRV can be evaluated through time-domain indices, such as the mean NN interval duration, frequency-domain measures based on spectral power in specific frequency ranges, and nonlinear approaches such as Sample Entropy and symbolic dynamics, which capture the complexity of heart rate dynamics [7,8].

Prior evidence links reduced HRV and autonomic imbalance with unfavorable outcomes in ischemic stroke. In a study by Beer et al., patients examined once in the subacute phase (three to six weeks post-stroke) demonstrated significantly lower HRV and impaired autonomic adaptability, characterized by sympathetic predominance and blunted parasympathetic modulation [9]. Similarly, Tobaldini et al. investigated autonomic dysfunction during the very early phase of acute ischemic stroke, defined as the emergency department presentation at stroke onset, and reported that higher sympathetic activity combined with reduced HRV was associated with unfavorable outcomes [10] and De Raedt et al. highlighted that sympathetic hyperactivity in ischemic stroke is linked to an unfavorable prognosis [11].

Despite these insights, important knowledge gaps remain. Prior studies have focused on a single time window or emphasized short-term HRV assessments, without fully characterizing the temporal evolution of HRV after stroke or its prognostic value across different recovery stages. Moreover, the extent to which the time course of autonomic recovery contributes to functional recovery and survival remains insufficiently clarified. In this study, we address some of these gaps by evaluating HRV at baseline (within 72 h), one month, and three months after acute ischemic stroke. In parallel with HRV measurements, functional outcome was assessed at the same time points using the modified Rankin Scale. Our aim is to determine the prognostic significance of HRV in both the acute and subacute phases, focusing on its association with functional recovery and mortality. By capturing temporal changes in HRV parameters and their relationship with clinical outcomes, our study offers additional insights into the role of autonomic recovery in post-stroke prognosis.

## 2. Materials and Methods

### 2.1. Population

We conducted a prospective observational study on a total of 148 patients diagnosed with AIS between January 2022 and October 2024. All patients were initially evaluated in the Emergency Department of the County Emergency Hospital “Pius Brinzeu” in Timișoara, Romania. AIS diagnostic was established based on clinical presentation and confirmed by computed tomography (CT) imaging.

Following diagnosis, patients were admitted to the Neurology Clinic, where each underwent a comprehensive clinical, biological, and paraclinical assessment. Management and treatment strategies were performed in accordance with the European Stroke Organisation (ESO) guidelines.

Baseline data collection included demographic characteristics, medical history, and laboratory parameters. For each patient, an initial assessment was conducted within the first 72 h of hospital admission (designated as the baseline), followed by follow-up assessments at one month and three months post-stroke.

HRV was assessed using 24-h Holter electrocardiogram (ECG) monitoring, and functional status was evaluated using the modified Rankin Scale (mRS) at all three time points (baseline, one month, and three months).

For the purposes of this study, the acute phase was defined as the first 72 h following stroke onset, when baseline HRV assessment was performed. The subacute phase refers to the period from one week to three months post-stroke, corresponding to the early rehabilitation period. Our follow-up evaluations at one month and three months therefore captured HRV dynamics within the subacute recovery phase.

**Figure 1:**
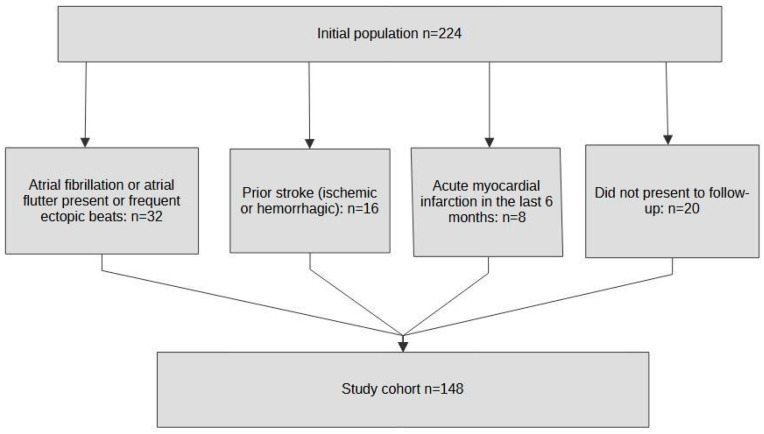
Flow diagram of study population selection.

Inclusion criteria comprised adult patients with confirmed AIS based on clinical evaluation and CT imaging. Patients were excluded if they had atrial fibrillation, atrial flutter, or frequent ectopic beats documented on Holter ECG recording; had a history of previous ischemic stroke or intracerebral hemorrhage; were diagnosed with acute myocardial infarction in the last six months; or did not present for follow-up assessment (Figure 1).

All included patients received appropriate treatment for stroke and associated comorbid conditions, in line with current clinical guidelines.

### 2.2. Heart Rate Variability Analysis

All patients underwent 24-h ECG monitoring using a Holter system. Holter ECG recordings were obtained at three predefined time points: baseline (within the first 72 h of hospital admission), one month, and three months following the onset of AIS.

The ECG recordings were analyzed to assess HRV, detect arrhythmic events, and identify additional electrical abnormalities. Data from the Holter monitors were transferred to a dedicated workstation and processed using the Labtech Cardiospy v5.03 (version 5.03.06.00, Labtech Ltd., Debrecen, Hungary) software.

All ECG recordings were subjected to manual inspection. Artifacts, ectopic beats, and periods of signal loss were identified visually and excluded using the filtering tools embedded in the Labtech Cardiospy v5.03 software, followed by manual verification to ensure data quality. Only segments with sinus rhythm were included in the analysis, in line with recommended standards for HRV measurement. HRV parameters were calculated from the 24-h recording to capture the global autonomic profile of each patient.

The time-domain parameter analyzed was SDNN, and frequency-domain analysis included LF power (0.04–0.15 Hz) and HF power (0.15–0.40 Hz). These spectral components were derived using either Fast Fourier Transform or autoregressive modeling, as implemented in the analysis software.

The primary aim of this study was to explore the association between HRV parameters derived from Holter ECG monitoring and functional outcomes, as measured by the mRS across the three time points.

### 2.3. Statistical Analysis

All analyses were performed in R Studio (version 2025.05.1 Build 513, Posit Software, PBC, Boston, MA, USA) with R (version 4.5.1). Continuous variables are reported as mean ± standard deviation (SD) if normally distributed and as median with interquartile range (IQR) if non-normally distributed. Categorical variables are presented as counts and percentages.

Normality of continuous variables was assessed separately at each time point using the Shapiro–Wilk test.

To analyze longitudinal changes in HRV parameters, we applied linear mixed-effects models (LMMs) with time as a fixed effect and patient ID as a random intercept. Because HRV parameters showed skewed distributions, all values were log-transformed prior to modeling, which substantially improved model fit according to Akaike Information Criterion (AIC) and Bayesian Information Criterion (BIC). For baseline and follow-up estimates, the results were back-transformed from the log scale to provide clinically interpretable values as absolute means with 95% confidence intervals, alongside relative percentage changes compared with baseline.

In addition, pairwise comparisons between timepoints—baseline to one month, baseline to three months, and one month to three months—were obtained from the same log-transformed linear mixed-effects model (log-LMM). These contrasts were back-transformed and reported as changes together with their standard errors and Bonferroni-adjusted *p*-values.

Continuous variables were compared between good outcome (mRS ≤ 2) and poor outcome (mRS > 2) groups at each time point. If both groups were normally distributed (Shapiro–Wilk *p* ≥ 0.05), Levene’s test was used to assess equality of variances, followed by Student’s *t*-test (equal variances) or Welch’s *t*-test (unequal variances). If either group was non-normal, the Mann–Whitney U test was applied. This approach allowed us to observe differences in absolute HRV parameters values between groups at specific time points, complementing the longitudinal trends described above.

Associations between continuous variables and ordinal mRS scores were evaluated using Spearman’s rank correlation. Spearman’s test was chosen to quantify unadjusted, monotonic associations between non-normally distributed HRV measures and the ordinal mRS across its range, without assuming linearity or interval scaling.

Binary logistic regression was used to identify predictors of dichotomized functional outcome (mRS ≤ 2 vs. mRS > 2), providing adjusted effect estimates and clinically interpretable odds ratios for the standard good–poor outcome benchmark. All multivariable models were checked for multicollinearity using the variance inflation factor (VIF), with VIF < 5 considered acceptable.

Mortality (mRS = 6) was assessed at baseline, one month, and three months, and rates were calculated for each follow-up. Associations between predictors and mortality at each timepoint were tested using *t*-tests or Mann–Whitney U tests for continuous variables (based on Shapiro–Wilk normality results) and Chi-square or Fisher’s exact tests for categorical variables, with resulting *p*-values summarized in a compact table and visualized as a heatmap to compare significance across timepoints.

A two-sided *p*-value < 0.05 was considered statistically significant for all analyses.

## 3. Results

### 3.1. Study Population Description

Table 1 shows that the mean age of the study cohort was 65.93 years (SD ± 9.19). Of the 148 participants, 54.05% were male, and 45.95% were female. The most prevalent comorbid condition was dyslipidemia, affecting 66.89% of the subjects, followed by arterial hypertension with 64.86% and diabetes mellitus with 47.30%.

Obesity and heart failure were both present in 38.51% of the study group. Additionally, coronary artery disease was documented in 35.14% of cases, and chronic kidney disease was reported in 24.32%. Regarding lifestyle related risk factors, 39.19% of the participants were current or former smokers, and 17.57% reported regular alcohol consumption.

When stratifying the cohort by functional outcome at baseline (good outcome—mRS ≤ 2 vs. poor outcome—mRS > 2), notable differences emerged between groups. Patients with poor outcomes were slightly older on average (67.94 ± 8.77 years vs. 64.84 ± 9.27 years) and showed a markedly higher prevalence of cardiovascular comorbidities, particularly heart failure (67.31% vs. 21.88%) and coronary artery disease (50.00% vs. 27.08%). Chronic kidney disease was also more frequent in the poor outcome group (30.77% vs. 20.83%). By contrast, obesity and dyslipidemia appeared somewhat more prevalent among patients with good outcomes (40.62% vs. 34.62% and 69.79% vs. 61.54%, respectively). Smoking and alcohol consumption were relatively balanced between groups.

### 3.2. HRV Analysis for the Entire Study Population

**Figure 2:**
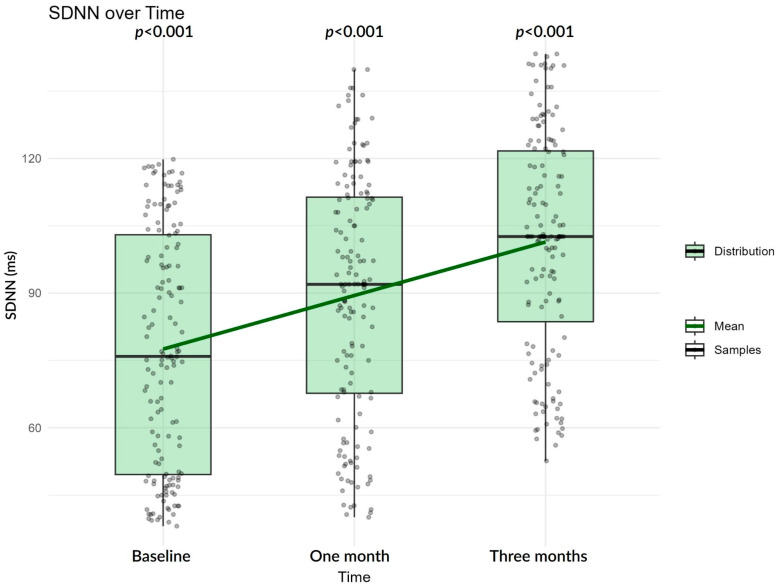
SDNN progression over time. Boxplots show the median and interquartile range with whiskers representing the data spread; individual gray dots indicate patient values; the green line connects group means across time points. Reported *p*-values (*p* < 0.001 for all intervals) were obtained from LMMs after log transformation.

**Figure 3:**
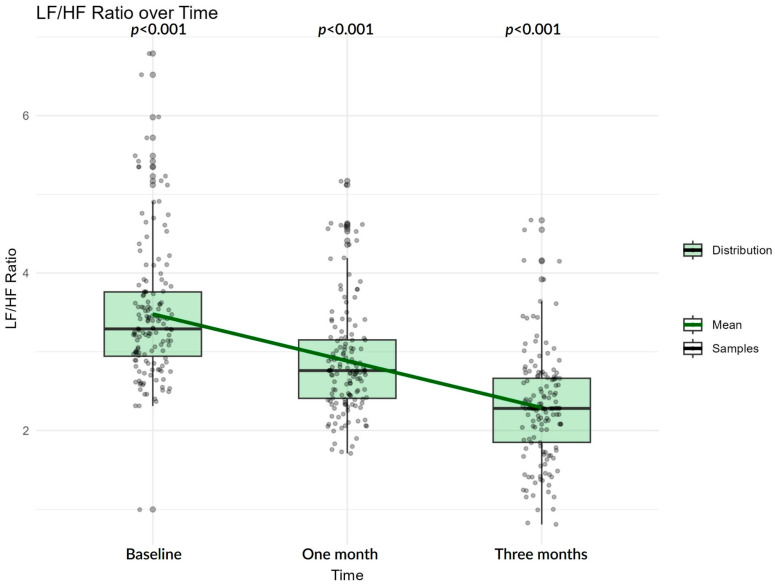
LF/HF progression over time. Boxplots show the median and interquartile range with whiskers representing the data spread; individual gray dots indicate patient values; the green line connects group means across time points. Reported *p*-values (*p* < 0.001 for all intervals) were obtained from LMMs after log transformation.

In the entire study cohort, a progressive increase in SDNN and decrease in LF/HF ratio is observed over the follow-up period, reflecting gradual recovery of overall heart rate variability. SDNN in average increases in a linear fashion, and median SDNN tends towards higher values (Figure 2), in contrast to LF/HF, which decreases (Figure 3), indicating improved autonomic adaptability.

These changes suggest a gradual shift in autonomic balance over time, which may reflect either reduced sympathetic activity, enhanced parasympathetic activity, or a combination of both. The LF/HF ratio showed a corresponding decline from 3.29 at baseline to 2.75 at one month and 2.30 at three months. Functional outcomes improved over follow-up, with median mRS decreasing from 2.00 at baseline to 1.00 at three months.

Table 2 presents the evolution of HRV parameters alongside functional status measured by the mRS at three time points: baseline, one month, and three months following AIS. Over the follow-up period, SDNN increased steadily from 75.90 ms at baseline to 91.95 ms at one month and 102.05 ms at three months, indicating a progressive recovery of overall autonomic modulation. This trend was accompanied by a decrease in LF power from 953.50 ms2 (baseline) to 782.00 ms2 (three months), and a rise in HF power from 282.41 ms2 (baseline) to 336.50 ms2 (three months).

The findings in Table 3 emphasize clinical interpretation, reporting baseline values together with changes in HRV. The results indicate that autonomic function, as reflected in HRV parameters, improves progressively during the first three months after stroke, with increased parasympathetic modulation and reduced sympathetic dominance.

At baseline, SDNN was 72.81 ms (95% CI: 69.10–76.71), increasing by 16.60% at one month and 34.84% at three months (*p* < 0.001 for both intervals), indicating a progressive recovery of overall autonomic modulation. LF power, initially 951.67 ms^2^, decreased by 5.19% at one month and 21.61% at three months (*p* < 0.001), while HF power rose from 278.82 ms^2^ to show a 15.30% increase at one month and a 22.26% increase at three months (*p* < 0.001), reflecting an increasing contribution of parasympathetic activity over time. The LF/HF ratio decreased significantly from 3.38 at baseline to show reductions of 16.91% at one month and 35.41% at three months (*p* < 0.001), consistent with a shift toward vagal predominance and improved sympathovagal balance.

The results in Table 4 focus on statistical contrasts reporting pairwise differences, and they show that SDNN increased steadily across all intervals, with a 16.60% rise from baseline to one month (estimate: 0.15, SE: 0.01, *p* < 0.001) and a 34.84% increase by three months (estimate: 0.29, SE: 0.01, *p* < 0.001). The additional 15.65% gain from one to three months underscores continued improvement in overall autonomic balance beyond the early recovery phase. LF power declined significantly over time. The largest reduction, −21.61%, was observed between baseline and three months (estimate: −0.24, SE: 0.02, *p* < 0.001). HF power exhibited significant gains, rising 15.30% (estimate: 0.14, SE: 0.02, *p* < 0.001) by one month and 22.26% (estimate: 0.20, SE: 0.02, *p* < 0.001) by three months. LF/HF ratio most pronounced decline was from baseline to three months by −35.41% (estimate: −0.43, SE: 0.02, *p* < 0.001).

The findings in Table 5 indicate that a higher SDNN and lower LF power in the early and subacute phases are associated with better functional outcomes at each follow-up visit.

LF/HF ratio was significantly lower in the good outcome group at baseline (*p* = 0.01) but showed no significant differences at later follow-ups.

Across all time points, SDNN was markedly higher in the good outcome group compared with the poor outcome group (*p* < 0.001). LF power was consistently lower in the good outcome group at all time points (*p* < 0.01), while HF power did not differ significantly between groups at any time point (*p* > 0.05 at all three time points).

Table 6 shows that, across all assessment points, SDNN presented a strong-to-moderate negative correlation with mRS, showing that a greater overall SDNN was associated with better functional status (baseline: Rho = −0.68, one month: Rho = −0.60, three months: Rho = −0.48; all *p* < 0.001). LF power demonstrated a consistent weak positive correlation with mRS (baseline: Rho = 0.32, one month: Rho = 0.30, three months: Rho = 0.26; all *p* < 0.001), whereas HF power exhibited very weak negative associations that became significant during follow-up (baseline: Rho = −0.12, *p* = 0.12; one month: Rho = −0.16, *p* = 0.03; three months: Rho = −0.18, *p* = 0.02). The LF/HF ratio maintained a weak positive correlation at all time points, highlighting that a more sympathetic-dominant autonomic profile was linked to a poorer functional recovery (baseline: Rho = 0.23, *p* < 0.01; one month: Rho = 0.32, *p* < 0.001; three months: Rho = 0.34, *p* < 0.001). mRS distribution shows that, at baseline, most patients clustered in the middle categories, with 35.10% scoring 2 and 20.30% scoring 3, with a median of 2 (IQR of 1–3, range of 0–4). By one month, the distribution shifted toward better outcomes, with a higher proportion achieving scores of 0–2 (73.60%), although 4.1% had died (mRS = 6); the median remained 2 (IQR of 1–3, range of 0–6). At three months, the improvement was more pronounced, with 76.30% achieving favorable scores of 0–2, the median decreasing to 1 (IQR of 0–2), and 12.20% classified as dead (mRS = 6).

Overall, the results presented in Table 7 demonstrate that HRV parameters, particularly a higher SDNN and lower LF/HF ratio, are consistent predictors of favorable functional recovery, even when adjusted for conventional cardiovascular risk factors.

At baseline, higher SDNN was strongly protective against poor outcome (OR 0.86, 95% CI 0.80–0.91, *p* < 0.001), indicating that, for each 1 ms increase in SDNN, the odds of poor outcome (mRS > 2) decreased by 14%. Conversely, a higher LF/HF ratio significantly increased the odds of poor outcome (OR 2.89, 95% CI 1.35–6.99, *p* = 0.01). Another finding at baseline indicated that heart failure was a significant predictor of poor outcome (OR 6.75, 95% CI 1.70–33.07, *p* = 0.01).

At one month, SDNN remained a significant protective factor (OR 0.91, 95% CI 0.86–0.94, *p* < 0.001), while the LF/HF ratio was not statistically associated with outcome (*p* = 0.21). Coronary artery disease was a strong predictor of poor outcome (OR 11.86, 95% CI 2.85–66.67, *p* < 0.001), and obesity appeared to be protective (OR 0.16, 95% CI 0.03–0.67, *p* = 0.02), aligning with reports of an “obesity paradox” in stroke prognosis.

At three months, a higher SDNN was again associated with a favorable outcome (OR 0.94, 95% CI 0.90–0.97, *p* < 0.001). The LF/HF ratio showed a significant positive association with poor outcome (OR 3.27, 95% CI 1.29–9.13, *p* = 0.02), suggesting that persistent sympathetic predominance may adversely affect long-term recovery.

### 3.3. Impact of HRV on Mortality Outcome

**Figure 4:**
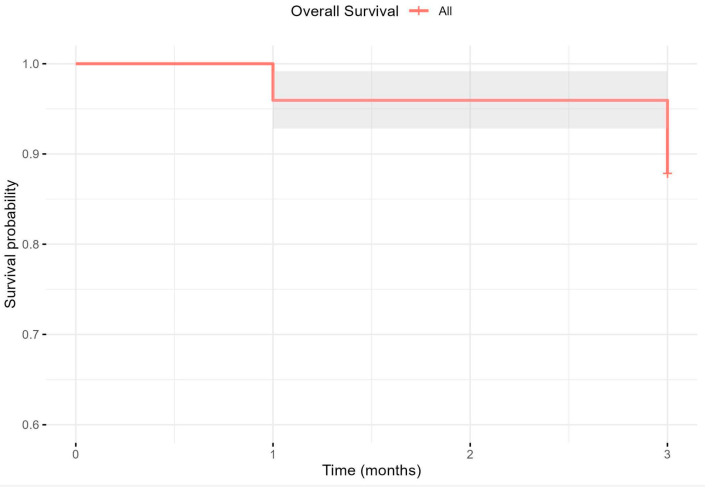
Survival probability over time. Red line represents the survival probability; grey area represents the confidence interval (95%).

Mortality was assessed for the entire study population. Figure 4 illustrates the temporal progression of mortality during follow-up. Survival declined stepwise to 95.90% at one month, and 87.80% at three months, with mortality increasing to 4.10% at one month and 12.20% at three months.

**Figure 5:**
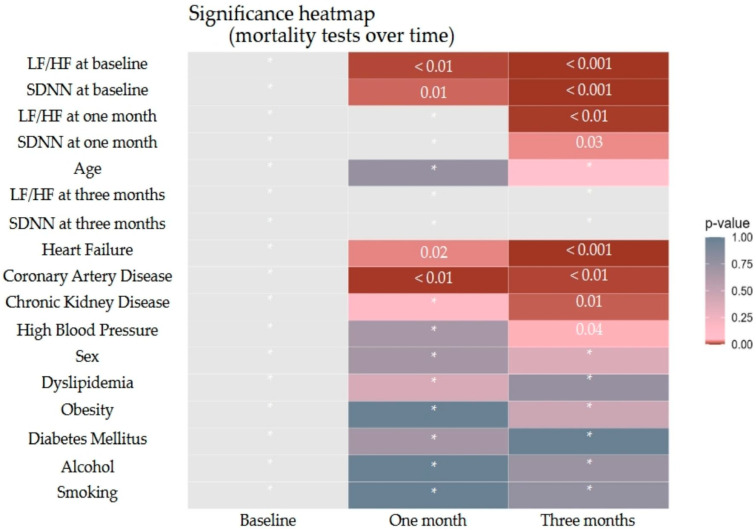
Mortality predictors. Cells marked with * represent non-significant results with *p* values greater than 0.05.

Analysis of potential risk factors revealed that both baseline comorbidities, particularly cardiovascular and renal diseases, and specific HRV parameters were significantly associated with mortality risk at different stages of follow-up, as illustrated in Figure 5.

At one month, the most significant predictors of mortality were the presence of coronary artery disease (Fisher’s exact test, *p* < 0.01), heart failure (Fisher’s exact test, *p* = 0.02), baseline LF/HF ratio (Mann–Whitney U-test, statistic = 150.50, *p* < 0.01), and baseline SDNN (Mann–Whitney U-test, statistic = 670.00, *p* = 0.01). By three months, mortality was significantly associated with coronary artery disease (Chi-square test, statistic = 7.43, *p* < 0.01), chronic kidney disease (Fisher’s exact test, *p* = 0.01), hypertension (Chi-square test, statistic = 4.05, *p* = 0.04), heart failure (Chi-square test, statistic = 12.03, *p* < 0.001), baseline LF/HF (Mann–Whitney U-test, statistic = 508.00, *p* < 0.001) and at one month (Mann–Whitney U-test, statistic = 656.50, *p* < 0.01), as well as SDNN at baseline (Mann–Whitney U-test, statistic = 1750.00, *p* < 0.001) and at one month (Mann–Whitney U-test, statistic = 1537.50, *p* = 0.03). These findings indicate that both pre-existing cardiovascular and renal comorbidities, together with autonomic dysfunction reflected by altered HRV parameters, contributed to increased mortality risk during follow-up, whereas other evaluated variables did not demonstrate statistically significant associations (*p* > 0.05).

## 4. Discussion

Our findings highlight that HRV parameters, particularly SDNN and the LF/HF ratio, serve as consistent predictors of functional recovery and mortality risk in patients with AIS during the first three months post-event. Progressive increases in SDNN and HF power, alongside marked reductions in LF power and LF/HF ratio, indicate a gradual shift from sympathetic dominance toward parasympathetic predominance as recovery progresses. Importantly, a lower baseline SDNN and higher LF/HF ratio were strongly associated with poor outcomes and increased mortality, emphasizing the prognostic significance of early autonomic dysfunction. These results suggest that impaired autonomic regulation characterized by sympathetic overactivity and reduced vagal tone may contribute to unfavorable neurological and survival outcomes. Consequently, early identification of HRV imbalances may offer a valuable opportunity to stratify risk, tailor rehabilitation strategies, and potentially target autonomic modulation as part of post-stroke care.

The mean age of our study population was 65.93 years (SD ± 9.19), aligning with previous evidence highlighting the strong association between advancing age and AIS. Approximately 75% of all strokes occur in individuals aged 65 years or older, emphasizing the heightened cerebrovascular vulnerability in this demographic. Increased clinical severity has been observed in AIS patients aged 65–74 years and especially in those ≥75 years, with the effect being more pronounced in the presence of comorbidities such as heart failure [12,13].

In our cohort, a high prevalence of cardiovascular and metabolic risk factors was evident. Heart failure, present in 38.51% of participants, is recognized as a strong determinant of stroke severity and unfavorable functional outcomes, largely due to its impact on cardiac output and cerebral perfusion, which can exacerbate ischemic injury [14]. Coronary artery disease, affecting 35.14% of the study population, similarly reflects the burden of atherosclerotic pathology contributing to cerebrovascular events.

Diabetes mellitus, documented in 47.30% of patients, is a well-established contributor to stroke risk through mechanisms involving endothelial dysfunction, accelerated atherosclerosis, and impaired neurovascular repair. As reported in the recent literature, diabetic patients often experience poorer recovery trajectories, with higher rates of post-stroke complications [15].

Other modifiable factors were present in our cohort, including CKD (24.32%), hypertension (64.86%), obesity (38.51%), smoking (39.19%), alcohol consumption (17.57%), and dyslipidemia (66.89%). These conditions are known to interact, contributing to vascular instability, heightened autonomic imbalance, and increased risk of adverse post-stroke outcomes [16,17].

In our study, obesity was present in 38.51% of the cohort and emerged as a protective factor against poor functional outcomes at 1 month. This aligns with the obesity paradox described in previous studies, suggesting that excess adiposity may provide metabolic reserves, modulate inflammatory responses, or confer cardiovascular resilience during the subacute recovery period. Doehner et al. found that overweight and obese patients with acute stroke or TIA had significantly better survival and improved functional status compared with their normal-weight counterparts [18]. Other studies further supported this observation, showing a linear association between higher BMI and increased survival in AIS [19,20].

Our results show that HRV parameters, particularly SDNN and LF/HF ratio, serve as strong predictors of clinical outcomes after AIS, at any time of measurement. Baseline lower SDNN values were consistently associated with increased mortality and poorer functional outcomes, reflecting a sustained state of sympathetic predominance and reduced vagal modulation. This aligns with prior observations that post-stroke patients with a markedly reduced SDNN exhibit persistent autonomic imbalance, indicative of ongoing sympathetic overactivity during the subacute phase when rehabilitation is typically initiated [11]. Autonomic imbalance not only compromises cardiovascular stability but also limits neuroplastic potential, thereby impeding neurological and motor recovery [21,22]. The prognostic relevance of HRV is further supported by findings from Xiong et al., who demonstrated that autonomic dysfunction independently predicted unfavorable functional outcomes at three months [23].

By assessing HRV longitudinally, our study reinforces the fact that lower SDNN and higher LF/HF ratios at baseline and one month remain strong indicators of adverse outcomes at three months. These temporal patterns suggest that not only the initial autonomic state but also the trajectory of recovery plays a critical role in prognosis. Patients with persistently low SDNN values and elevated LF/HF ratios throughout the follow-up period had markedly poorer survival and functional recovery rates. This is consistent with the concept that early autonomic impairment continues to exert a detrimental influence over time [23]. While Kuo et al. reported that elevated mean in-hospital heart rate in the first three days after AIS was linked to worse three month outcomes [24], our findings extend this by showing that autonomic regulation reflected by both SDNN and LF/HF can track recovery progression and signal prognosis well beyond the acute phase.

Our study also highlights the prognostic value of HRV parameters for stratifying outcomes in AIS. Patients with higher SDNN values and lower LF/HF ratios consistently demonstrated better functional recovery and lower mortality rates. Conversely, those with depressed SDNN at both baseline and one month follow-up exhibited significantly higher mortality risk and were more likely to have poor functional outcomes at three months. This pattern aligns with prior work by Sethi et al. [21] and Scherbakov et al. [22], which showed that higher HRV at admission predicted improved motor recovery, and with Wu et al. [25], who linked autonomic imbalance to both functional decline and mortality risk. The persistent sympathetic predominance reflected in elevated LF/HF ratios may contribute to increased cardiovascular strain, impaired neurovascular repair, and reduced adaptability to physiological stressors [11].

Regarding comorbidities, our mortality analyses identified CAD, HF, CKD, and high blood pressure as significant contributors at one and/or three months. Prior studies document autonomic imbalance in acute stroke and its interaction with cardiovascular burden [26,27]. Studies relating elevated early heart rate and reduced HRV to worse 90-day outcomes further suggest that sympathetic overactivity is a shared pathway connecting comorbidity load and post-stroke mortality [24,28]. This supports our conclusion that HRV markers (higher SDNN, lower LF/HF) track recovery and survival, while cardiovascular and renal comorbidities amplify mortality risk through sustained autonomic imbalance.

We observed that patients who showed greater gains in SDNN and a steady decline in LF/HF over time tended to recover better. This pattern suggests that restoring autonomic balance by improving parasympathetic activity may play an important role in supporting functional recovery and outcome during the subacute phase. Similar findings have been reported by Wu et al. [25] and Li et al. [29], where HRV measures were identified as promising markers for predicting functional outcomes at three months.

The added benefit of our study lies in its longitudinal evaluation of HRV across the acute and subacute phases of AIS, enabling us to show that not only baseline autonomic dysfunction but also the trajectory of autonomic recovery carries prognostic significance. By assessing HRV at three distinct time points, we highlight that patients with progressive restoration of parasympathetic activity, reflected in rising SDNN and declining LF/HF ratios, achieve better functional recovery and survival compared with those in whom sympathetic predominance persists. This dynamic perspective extends beyond prior cross-sectional work by illustrating that the direction and magnitude of change in HRV parameters provide added insight. This trend may improve prognosis because sympathetic downregulation reduces blood pressure variability and cardiac strain, while enhanced parasympathetic activity supports cardiovascular stability and facilitates neuroplastic recovery.

Our study has several limitations. As a first point, this was a single center investigation, and as such should be interpreted with caution when applied to different clinical settings or populations. Second, our sample size (N = 148), while adequate for detecting significant associations, may still limit the statistical power for detailed subgroup analyses, particularly when examining interactions between specific comorbidities and HRV parameters. Third, the cohort had a mean age of 65.93 years, with a predominance of older adults, which may restrict generalization to younger stroke populations.

Another limitation is that patients with an mRS score of 5 at baseline were excluded, as their severe disability made consistent evaluation across repeated follow-up visits impractical. This may have led to an underrepresentation of the most severely affected patients, potentially biasing the associations toward better outcomes.

Additionally, although HRV parameters were systematically assessed at baseline, one month, and three months, potential confounders such as administration of autonomic modulating agents and their timing relative to measurements were not captured, which could influence HRV readings. Moreover, while we observed significant longitudinal changes in HRV parameters and their associations with mortality and functional outcome, we did not assess other potential contributors to autonomic dysfunction such as sleep quality, physical activity levels during recovery, or emotional stress factors that could modulate HRV over time.

In our study, some patients initially classified in the poor-outcome group transitioned into the good-outcome group during follow-up, while very few showed the opposite trajectory. Future work should examine whether specific baseline features or comorbidities predict recovery or deterioration.

Future research should expand on our findings by including larger, multicenter cohorts with more diverse age groups and incorporating detailed medication and lifestyle data. Longitudinal follow-up beyond three months would be valuable to determine whether early changes in HRV parameters predict sustained survival and functional recovery.

## 5. Conclusions

Our study indicates that specific HRV parameters, particularly lower SDNN value and higher LF/HF ratio at baseline and early follow-up, are important predictors of mortality and poor functional outcomes after AIS. Both diminished vagal modulation and sustained sympathetic predominance were consistently associated with increased risk of death and unfavorable recovery, regardless of other clinical factors. Mortality risk was the highest among patients with persistently depressed SDNN and elevated LF/HF ratios, while those with greater improvement in autonomic balance over time showed better survival and recovery outcome.

These findings support the prognostic importance of evaluating HRV in the acute and subacute phases of AIS and suggest that targeted strategies to restore autonomic balance may offer therapeutic potential.

## Figures and Tables

**Table 1:** Study population description.

Characteristics	Values, N = 148 (%)	Good Outcome at Baseline, N = 96 (%)	Poor Outcome at Baseline, N = 52 (%)
Age	65.93 (9.19) *	64.84 (9.27) *	67.94 (8.77) *
Sex	M: 54.05 F: 45.95	M: 51.04 F: 48.96	M: 59.62 F: 40.38
Heart Failure	38.51	21.88	67.31
High Blood Pressure	64.86	64.58	65.38
Coronary Artery Disease	35.14	27.08	50.00
Diabetes Mellitus	47.30	44.79	51.92
Chronic Kidney Disease	24.32	20.83	30.77
Obesity	38.51	40.62	34.62
Dyslipidemia	66.89	69.79	61.54
Alcoholic	17.57	17.71	17.31
Smoker	39.19	36.46	44.23

* = Age expressed as mean value and standard deviation in the form of Mean (SD); Good outcome = patients with mRS ≤ 2 at baseline; Poor outcome = patients with mRS > 2 at baseline.

**Table 2:** Temporal changes in HRV parameters (SDNN, LF power, HF power, LF/HF ratio) and functional outcome (mRS) at baseline, one month, and three months.

Parameter	Time Point	Median (IQR)	Mean (SD)	Min–Max
SDNN	Baseline	75.90 (49.60–103.00)	77.53 (26.41)	38.10–119.80
	One month	91.95 (67.68–112.20)	89.86 (27.70)	40.10–139.80
	Three months	102.05 (77.03–122.40)	100.7 (25.98)	49.65–148.07
LF power	Baseline	953.50 (864.00–1036.00)	959.93 (127.95)	735–1291
	One month	889.00 (806.25–1023.00)	912.42 (143.14)	629–1293
	Three months	782.00 (670.25–897.50)	772.80 (190.20)	267–1245
HF power	Baseline	282.41 ± 44.16	282.41 (44.16)	189–401
	One month	327.00 (295.00–355.25)	326.04 (50.66)	209–499
	Three months	336.50 (298.50–386.25)	351.76 (96.41)	130–930
LF/HF	Baseline	3.29 (2.94–3.76)	3.48 (0.87)	1.00–6.79
	One month	2.75 (2.40–3.17)	2.88 (0.71)	1.71–5.17
	Three months	2.30 (1.84–2.70)	2.32 (0.71)	0.81–4.67
mRS	Baseline	2.00 (1.00–3.00)	2.15 (1.11)	0–4
	One month	2.00 (1.00–3.00)	1.85 (1.42)	0–6
	Three months	1.00 (0.00–2.00)	1.68 (1.70)	0–6

HRV = Heart Rate Variability; SDNN = Standard Deviation of NN intervals measured in milliseconds (ms); LF = Low-Frequency power measured in milliseconds squared (ms^2^); HF = High-Frequency power measured in milliseconds squared (ms^2^); IQR = Inter-Quartile Range; SD = Standard Deviation; mRS = modified Rankin Scale.

**Table 3:** Estimated baseline values and back-transformed percentage changes in HRV parameters from baseline to one and baseline to three months after acute ischemic stroke (N = 148).

Parameter	Time	Estimate	95% CI (Lower–Upper)	*p*	Change%
SDNN	Baseline	72.81	69.10–76.71	<0.001	
	Baseline to one month	1.17	1.13–1.20	<0.001	16.60
	Baseline to three months	1.35	1.31–1.39	<0.001	34.84
LF power	Baseline	951.67	922.05–982.24	<0.001	
	Baseline to one month	0.95	0.92–0.98	<0.001	−5.19
	Baseline to three months	0.78	0.76–0.81	<0.001	−21.61
HF power	Baseline	278.82	270.65–287.25	<0.001	
	Baseline to one month	1.15	1.11–1.19	<0.001	15.30
	Baseline to three months	1.22	1.18–1.27	<0.001	22.26
LF/HF	Baseline	3.38	3.24–3.53	<0.001	
	Baseline to one month	0.83	0.79–0.87	<0.001	−16.91
	Baseline to three months	0.65	0.62–0.68	<0.001	−35.41

HRV = Heart Rate Variability; SDNN = Standard Deviation of NN intervals measured in milliseconds (ms); LF = Low-Frequency power measured in milliseconds squared (ms^2^); HF = High-Frequency power measured in ms^2^; CI = Confidence Interval; Change% corresponds to (Estimate − 1) × 100. Explanatory notes: For baseline rows, estimate values represent raw device-acquired means with 95% confidence intervals, and associated *p*-values are from one-sample tests against zero. Baseline-to-follow-up values are obtained from log-transformed linear mixed-effects model; estimates were then back-transformed to the original scale and expressed as ratios relative to baseline, where values > 1 indicate increases and <1 indicate decreases relative to baseline.

**Table 4:** Pairwise log-scale contrasts of HRV parameter changes between timepoints following acute ischemic stroke (N = 148).

Parameter	Time	Estimate	SE	*p*	Change%
SDNN	Baseline to one month	0.15	0.01	<0.001	16.60
	Baseline to three months	0.29	0.01	<0.001	34.84
	One month to three months	0.14	0.01	<0.001	15.65
LF power	Baseline to one month	−0.05	0.02	<0.01	−5.19
	Baseline to three months	−0.24	0.02	<0.001	−21.61
	One month to three months	−0.19	0.02	<0.001	−17.32
HF power	Baseline to one month	0.14	0.02	<0.001	15.30
	Baseline to three months	0.20	0.02	<0.001	22.26
	One month to three months	0.05	0.02	<0.001	6.03
LF/HF	Baseline to one month	−0.18	0.02	<0.001	−16.91
	Baseline to three months	−0.43	0.02	<0.001	−35.41
	One month to three months	−0.25	0.02	<0.001	−22.27

HRV = Heart Rate Variability; SDNN = Standard Deviation of NN intervals measured in milliseconds (ms); LF = Low-Frequency power measured in milliseconds squared (ms^2^); HF = High-Frequency power measured in milliseconds squared (ms^2^); Estimate represents log-scale pairwise contrasts from log-LMM: positive values indicate increases and negative values decreases relative to the earlier time point; SE = Standard Error, showing sampling uncertainty of the Estimate; Change% is the back-transformed percentage change corresponding to the log-scale Estimate, reflecting percentage change in the original Parameter.

**Table 5:** Comparison of HRV parameters between patients with good and poor functional outcomes at baseline, one month, and three months after acute ischemic stroke (N = 148).

Parameter	Time	Good Outcome (mRS ≤ 2)	Poor Outcome (mRS > 2)	Good Outcome Median (IQR)	Poor Outcome Median (IQR)	*p*
SDNN	Baseline	N = 96	N = 52	92.60 (33.85)	48.20 (12.57)	<0.001
LF power				930.50 (183.00)	987.00 (169.75)	<0.01
HF power				286.00 (60.75)	279.50 (61.00)	0.06
LF/HF				3.27 (0.76)	3.41 (1.20)	0.01
SDNN	One month	N = 108	N = 40	99.30 (29.30)	56.65 (25.00)	<0.001
LF power				886.50 (218.25)	1000.50 (175.50)	<0.01
HF power				333.00 (63.50)	327.00 (44.25)	0.18
LF/HF				2.72 (0.82)	2.80 (0.68)	0.16
SDNN	Three months	N = 113	N = 35	109.70 (34.40)	65.30 (0.15)	<0.001
LF power				772.00 (222.00)	267.00 (590.00)	<0.01
HF power				336.00 (87.00)	355.00 (18.00)	0.72
LF/HF				2.20 (0.99)	2.08 (0.45)	0.35

HRV = Heart Rate Variability; SDNN = Standard Deviation of NN intervals measured in milliseconds (ms); LF = Low-Frequency power measured in milliseconds squared (ms^2^); HF = High-Frequency power measured in milliseconds squared (ms^2^); IQR = Inter-Quartile Range; mRS = modified Rankin Scale.

**Table 6:** Correlation between HRV parameters and functional outcome (mRS) at baseline, one month, and three months after acute ischemic stroke, with mRS distribution at each time point (N = 148).

Correlations Between HRV Parameters			
Parameter	Time	Rho	*p*
SDNN	Baseline	−0.68	<0.001
LF power		0.32	<0.001
HF power		−0.12	0.12
LF/HF		0.23	<0.01
SDNN	One month	−0.60	<0.001
LF power		0.30	<0.001
HF power		−0.16	0.03
LF/HF		0.32	<0.001
SDNN	Three months	−0.48	<0.001
LF power		0.26	<0.001
HF power		−0.18	0.02
LF/HF		0.34	<0.001
**mRS Distribution Spread**			
**Time point**	**Distribution** **mRS: Count (%)**		
Baseline	0: 8 (5.40%), 1: 36 (24.30%), 2: 52 (35.10%), 3: 30 (20.30%), 4: 22 (14.90%), 5: 0 (0%), 6: 0 (0%); Median 2 (IQR of 1–3); Range of 0–4		
One month	0: 26 (17.60%), 1: 37 (25.00%), 2: 45 (30.40%), 3: 25 (16.90%), 4: 9 (6.10%), 5: 0 (0%), 6: 6 (4.10%); Median 2 (IQR of 1–3), Range of 0–6		
Three months	0: 40 (27%), 1: 37 (25%), 2: 36 (24.30%), 3: 14 (9.50%), 4: 3 (2%), 5: 0 (0%), 6: 18 (12.20%); Median 1 (IQR of 0–2), Range of 0–6		

HRV = Heart Rate Variability; SDNN = Standard Deviation of NN intervals measured in milliseconds (ms); LF = Low-Frequency power measured in milliseconds squared (ms^2^); HF = High-Frequency power measured in milliseconds squared (ms^2^); Rho = Spearman correlation coefficient; mRS = modified Rankin Scale; IQR = Inter-Quartile Range. Explanatory note: Top part shows correlations between HRV parameters; bottom part shows mRS distribution spread.

**Table 7:** Multivariable logistic regression analysis of predictors of poor functional outcome (mRS > 2) at baseline, one month, and three months after acute ischemic stroke (N = 148).

	Baseline		One Month		Three Month	
Parameter	OR (95% CI)	*p*	OR (95% CI)	*p*	OR (95% CI)	*p*
SDNN	0.86 (0.80–0.91)	**<0.001**	0.91 (0.86–0.94)	**<0.001**	0.94 (0.90–0.97)	**<0.001**
LF/HF	2.89 (1.35–6.99)	**0.01**	1.92 (0.73–5.70)	0.21	3.27 (1.29–9.13)	**0.02**
Age	1.02 (0.95–1.11)	0.54	1 (0.93–1.08)	0.99	1.02 (0.96–1.09)	0.5
Sex	2.9 (0.67–14.87)	0.17	1.05 (0.27–4.27)	0.94	1.47 (0.48–4.8)	0.51
Alcohol	1.24 (0.16–12.04)	0.84	2.84 (0.39–24.14)	0.31	2.84 (0.68–12.6)	0.16
Smoking	2.82 (0.68–13.63)	0.17	3.8 (0.88–19.64)	0.09	1.41 (0.44–4.68)	0.56
Diabetes Mellitus	0.75 (0.18–2.95)	0.68	0.96 (0.27–3.43)	0.96	0.71 (0.24–2.03)	0.52
Coronary Artery Disease	2.24 (0.53–10.82)	0.28	11.86 (2.85–66.67)	**<0.001**	2.65 (0.88–8.29)	0.08
Chronic Kidney Disease	0.98 (0.22–4.43)	0.98	3.66 (0.97–15.26)	0.06	2.2 (0.70–7.14)	0.18
High Blood Pressure	1.1 (0.29–4.44)	0.89	0.46 (0.12–1.65)	0.25	1.88 (0.62–5.97)	0.27
Heart Failure	6.75 (1.70–33.07)	**0.01**	3.25 (0.93–12.54)	0.07	2.33 (0.80–7.15)	0.13
Obesity	0.24 (0.03–1.24)	0.11	0.16 (0.03–0.67)	**0.02**	0.33 (0.09–1.09)	0.08
Dyslipidemia	0.31 (0.06–1.4)	0.14	0.23 (0.05–1.01)	0.06	2.13 (0.63–7.74)	0.23

mRS = modified Rankin Scale; SDNN = Standard Deviation of NN intervals measured in milliseconds (ms); LF = Low-Frequency power measured in milliseconds squared (ms^2^); HF = High-Frequency power measured in milliseconds squared (ms^2^); OR = Odds Ratio; CI = Confidence Interval. Explanatory note: statistically significant *p* values are highlighted in bold.

## Data Availability

Data are contained within the article.

## References

[1] Katan M., Luft A. (2018). Global Burden of Stroke. Semin. Neurol..

[2] United Nations (2007). World Population Ageing 2007.

[3] Damkjær M., Simonsen S.A., Heiberg A.V., Andersen L., Winther K., Hjort N., Johnsen S.P., Møller A.M., Iversen H.K., Christensen H. (2023). Autonomic Dysfunction after Mild Acute Ischemic Stroke and Six Months after: A Prospective Observational Cohort Study. BMC Neurol..

[4] Tenberg A., Tahara N., Grewal A., Holwerda S., Jackson C.A., Lattanzi S., Lee H., Leigh R., Nakanishi K., Rothwell P.M. (2024). Dysautonomia and Activity in the Early Stroke Recovery Period. Neurol. Sci..

[5] Armstrong R., Wheen P., Brandon L., Maree A., Kenny R.-A. (2022). Heart Rate: Control Mechanisms, Pathophysiology and Assessment of the Neurocardiac System in Health and Disease. QJM.

[6] Bogdan C., Apostol A., Ivan V.M., Sandu O.E., Petre I., Suciu O., Marc L.-E., Maralescu F.-M., Lighezan D.F. (2024). Heart Rate Variability and Global Longitudinal Strain for Prognostic Evaluation and Recovery Assessment in Conservatively Managed Post-Myocardial Infarction Patients. J. Clin. Med..

[7] Aftyka J., Staszewski J., Dębiec A., Pogoda-Wesołowska A., Żebrowski J. (2023). Can HRV Predict Prolonged Hospitalization and Favorable or Unfavorable Short-Term Outcome in Patients with Acute Ischemic Stroke?. Life.

[8] Task Force of the European Society of Cardiology and the North American Society of Pacing and Electrophysiology (1996). Heart Rate Variability. Circulation.

[9] Beer R.N., Soroker N., Bornstein N.M., Katz-Leurer M. (2018). The Cardiac Autonomic Nervous System Response to Different Daily Demands among Patients at the Sub-Acute Phase Post Ischemic Stroke and Healthy Controls. NeuroRehabilitation.

[10] Tobaldini E., Sacco R.M., Serafino S., Tassi M., Gallone G., Solbiati M., Costantino G., Montano N., Torgano G. (2019). Cardiac Autonomic Derangement Is Associated with Worse Neurological Outcome in the Very Early Phases of Ischemic Stroke. J. Clin. Med..

[11] De Raedt S., De Vos A., De Keyser J. (2015). Autonomic Dysfunction in Acute Ischemic Stroke: An Underexplored Therapeutic Area?. J. Neurol. Sci..

[12] Li L., Scott C.A., Rothwell P.M. (2022). Association of Younger vs Older Ages with Changes in Incidence of Stroke and Other Vascular Events, 2002–2018. JAMA.

[13] Edrissi C., Rathfoot C., Knisely K., Sanders C.B., Goodwin R., Nathaniel S.I., Nathaniel T. (2023). Age Stratification in Acute Ischemic Stroke Patients with Heart Failure. J. Clin. Med..

[14] Scherbakov N., Haeusler K.G., Doehner W. (2015). Ischemic Stroke and Heart Failure: Facts and Numbers. ESC Heart Fail..

[15] Sacco S., Foschi M., Ornello R., De Santis F., Pofi R., Romoli M. (2024). Prevention and Treatment of Ischaemic and Haemorrhagic Stroke in People with Diabetes Mellitus: A Focus on Glucose Control and Comorbidities. Diabetologia.

[16] Schumacher K., Kornej J., Shantsila E., Lip G.Y.H. (2018). Heart Failure and Stroke. Curr. Heart Fail. Rep..

[17] Jiang Y., Han J., Spencer P., Li Y., Vodovoz S.J., Ning M.-M., Liu N., Wang X., Dumont A.S. (2021). Diabetes Mellitus: A Common Comorbidity Increasing Hemorrhagic Transformation after tPA Thrombolytic Therapy for Ischemic Stroke. Brain Hemorrhages.

[18] Doehner W., Schenkel J., Anker S.D., Springer J., Audebert H.J. (2013). Overweight and Obesity Are Associated with Improved Survival, Functional Outcome, and Stroke Recurrence after Acute Stroke or Transient Ischaemic Attack: Observations from the TEMPiS Trial. Eur. Heart J..

[19] Chaudhary D., Khan A., Gupta M., Hu Y., Li J., Abedi V., Zand R. (2021). Obesity and Mortality after the First Ischemic Stroke: Is Obesity Paradox Real?. PLoS ONE.

[20] Liu Z., Sanossian N., Starkman S., Avila-Rinek G., Eckstein M., Sharma L.K., Liebeskind D., Conwit R., Hamilton S., Saver J.L. (2021). Adiposity and Outcome after Ischemic Stroke. Stroke.

[21] Sethi A., Callaway C.W., Sejdić E., Terhorst L., Skidmore E.R. (2016). Heart Rate Variability Is Associated with Motor Outcome 3-Months after Stroke. J. Stroke Cerebrovasc. Dis..

[22] Scherbakov N., Barkhudaryan A., Ebner N., von Haehling S., Anker S.D., Joebges M., Doehner W. (2020). Early Rehabilitation after Stroke: Relationship between the Heart Rate Variability and Functional Outcome. ESC Heart Fail..

[23] Xiong L., Tian G., Leung H., Soo Y.O.Y., Chen X., Ip V.H.L., Mok V.C.T., Chu W.C.W., Wong K.S., Leung T.W.H. (2018). Autonomic Dysfunction Predicts Clinical Outcomes after Acute Ischemic Stroke. Stroke.

[24] Kuo Y.-W., Lee M., Huang Y.-C., Lee J.-D. (2021). Initial In-Hospital Heart Rate Is Associated with Three-Month Functional Outcomes after Acute Ischemic Stroke. BMC Neurol..

[25] Wu M.-J., Dewi S.R.K., Hsu W.-T., Hsu T.-Y., Liao S.-F., Chan L., Lin M.-C. (2024). Exploring Relationships of Heart Rate Variability, Neurological Function, and Clinical Factors with Mortality and Behavioral Functional Outcome in Patients with Ischemic Stroke. Diagnostics.

[26] Yperzeele L., van Hooff R.J., Nagels G., De Smedt A., De Keyser J., Brouns R. (2015). Heart Rate Variability and Baroreceptor Sensitivity in Acute Stroke: A Systematic Review. Int. J. Stroke.

[27] Lago S., de Beukelaar T.T., Casetta I., Arcara G., Mantini D. (2025). Heart Rate Variability and Autonomic Dysfunction After Stroke: Prognostic Markers for Recovery. Biomedicines.

[28] Qu Y., Sun Y.-Y., Abuduxukuer R., Si X.-K., Zhang P., Ren J.-X., Fu Y.-L., Zhang K.-J., Liu J., Zhang P.-D. (2023). Heart Rate Variability Parameter Changes in Patients with Acute Ischemic Stroke Undergoing Intravenous Thrombolysis. J. Am. Heart Assoc..

[29] Li C., Meng X., Pan Y., Li Z., Wang M., Wang Y. (2021). The Association Between Heart Rate Variability and 90-Day Prognosis in Patients with Transient Ischemic Attack and Minor Stroke. Front. Neurol..

